# *In-vivo* biomagnetic characterisation of the American cockroach

**DOI:** 10.1038/s41598-018-23005-1

**Published:** 2018-03-23

**Authors:** Ling-Jun Kong, Herbert Crepaz, Agnieszka Górecka, Aleksandra Urbanek, Rainer Dumke, Tomasz Paterek

**Affiliations:** 1 School of Physical and Mathematical Sciences, Nanyang Technological University, Singapore, 637371 Singapore; 2 MOE Key Laboratory of Weak Light Nonlinear Photonics and School of Physics, Nankai University, Tianjin, 300071 China; 3 Centre for Quantum Technologies, National University of Singapore, Singapore, 117543 Singapore; 4 School of Physics and Astronomy, Monash University, Melbourne, 3800 Australia; 5 Department of Invertebrate Zoology and Parasitology, University of Gdańsk, Gdańsk, 80-308 Poland

## Abstract

We present a quantitative method, utilising a highly sensitive quantum sensor, that extends applicability of magnetorelaxometry to biological samples at physiological temperature. The observed magnetic fields allow for non-invasive determination of physical properties of magnetic materials and their surrounding environment inside the specimen. The method is applied to American cockroaches and reveals magnetic deposits with strikingly different behaviour in alive and dead insects. We discuss consequences of this finding to cockroach magneto-reception. To our knowledge, this work represents the first characterisation of the magnetisation dynamics in live insects and helps to connect results from behavioural experiments on insects in magnetic fields with characterisation of magnetic materials in their corpses.

## Introduction

Many species are capable of perceiving the world through senses inaccessible to humans. Polarisation vision of marine species^1^ or magnetic field detection by migratory birds^2^ being two well-known examples. Magneto-reception is in fact common to a wide range of organisms, ranging from bacteria to higher vertebrates, and has evolved to a fine-tuned sensory system that maybe even takes advantage of quantum coherence^3^. Insights into magneto-reception mechanisms and biomagnetism, magnetic fields that originate in biological systems, not only allow us to understand better different ways of visualising the world but may also find applications in improved man-made sensors inspired by their biological counterparts.

Several behavioural experiments have demonstrated that cockroaches and other insects are capable of magneto-reception, see e.g.^4–17^. Most of the behavioural experiments on cockroaches show their ability to perceive changes of an Earth-strength magnetic field that is rotated with a period of 10 minutes. A different set of experiments found and characterised magnetic particles in insect corpses, see e.g.^18–25^. Naturally one asks if these magnetic particles contribute to magneto-reception. One route to answer this question is to develop techniques for tracking dynamics and quantifying the extent of magnetic materials inside a living insect.

Here we demonstrate a non-invasive method for magnetic field measurements taking advantage of the high precision of atomic magnetometer^26^. It is a form of magnetorelaxometry (MRX)^27^ where the magnetic materials to be characterised could be present inside a living organism. The method is applied to study magnetic fields generated by magnetised American cockroaches (*Periplaneta americana*). Our study reveals presence of magnetic materials in their bodies exhibiting distinct dynamics in alive and dead cockroaches. After magnetisation of alive insects, we observe exponential magnetic field decay to a remnant value, with a decay time of 50 ± 28 minutes. In contradistinction, an average demagnetisation of dead cockroaches displays a much longer decay time of 47.5 ± 28.9 hours.

This clear difference in magnetic field decay is explained by Brownian rotations of magnetic materials in different viscosity environments. Fits of this model to the measured data reveal magnetic particles embedded in an environment which experiences a two orders of magnitude viscosity increment between alive and dead cockroaches. Under the assumption that there were no external magnetic particles on measured insects we find sub-micron sized magnetic deposits and a glassy environment of high viscosity. Such deposits would require several hours to align with an Earth-strength magnetic field. Thus, they cannot be responsible for the observed cockroach magneto-reception in rotating magnetic fields with a period of 10 minutes^4–7^. This provides further support to additional working mechanism behind magneto-reception, e.g. the radical-pair mechanism^6,7^.

## Results

**Figure 1:**
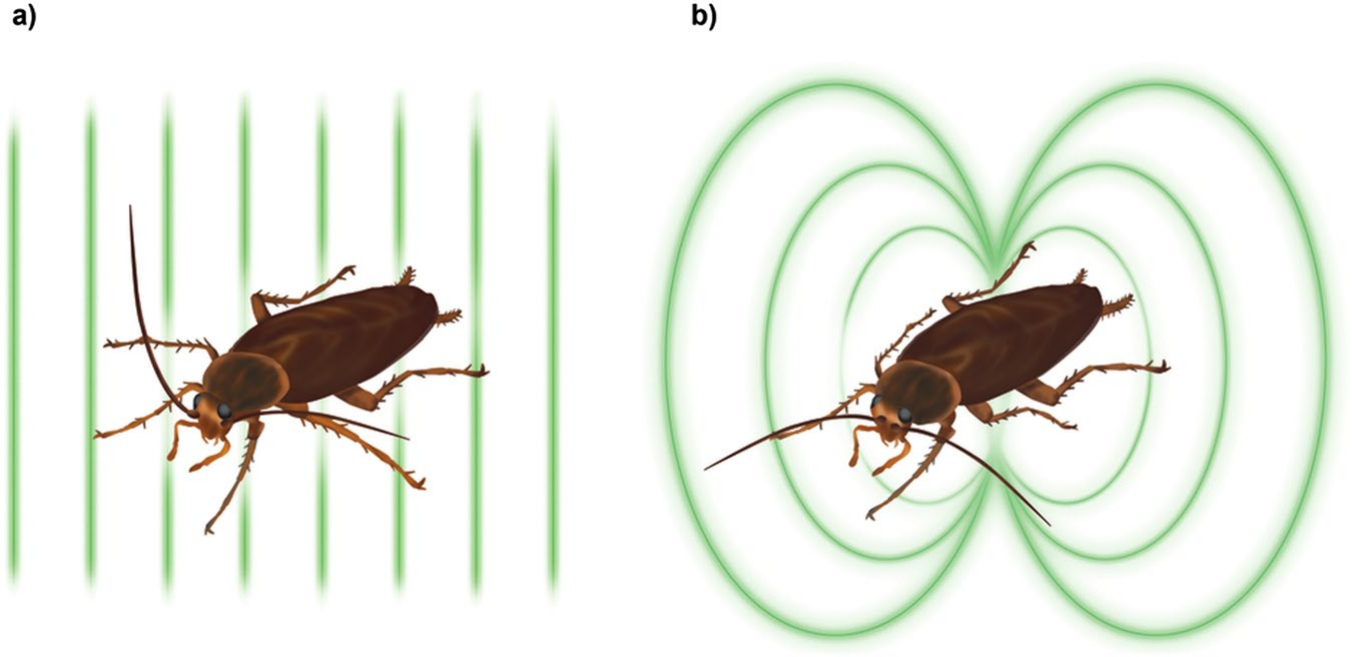
Sketch of the experiment. (**a**) American cockroaches were placed in a strong magnetic field aligned perpendicular to the thorax as illustrated by the green lines. Using an atomic magnetometer we monitored the dynamics of the magnetic field generated by the magnetised insects. (**b**) The magnetic field is very close to the field of magnetic dipole normal to the thorax. Published with permission from T. Yeo.

**Figure 2:**
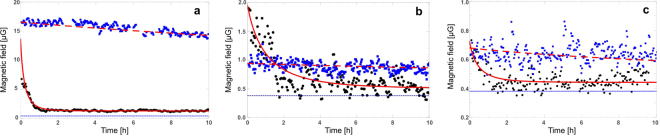
Magnetic field decay from magnetised American cockroaches. Black dots show the measured time dependence of the magnetic field for alive cockroaches and blue squares show this dependence for the dead ones. Different panels present exemplary data for different insects. They were chosen to show the typical sets where the initial magnetisation of alive cockroach can be higher, similar or lower than the initial magnetisation of the dead one. This is captured in our model as well. Altogether we conducted 15 measurements lasting longer than 10 hours each and additionally more than 10 shorter measurements. The thick red lines represent simulation of our model, fitting the data: solid for alive cockroaches and dashed for dead ones. The exponential decay time of the magnetic field is (**a**) 25 mins [82.6 hours], (**b**) 71 mins [36.3 hours], (**c**) 30 mins [24 hours] for alive [dead] cockroach. The average exponential decay time over all measurements is 50 ± 28 mins (47.5 ± 28.9 hours) for alive (dead) cockroaches. The offset magnetisation of 0.38 *μ*G (thin dashed line) is attributed to the cockroach container dominating the signal for unmagnetised cockroaches. Each data-point has an uncertainty of 0.08 *μ*G. Note different vertical scales in different panels.

The general idea of our experiments is depicted in Fig. 1. Our setup and detailed methodology are described in the Methods section. In short, magnetised cockroaches were measured with an all-optical Caesium atomic magnetometer with periodically moved sample tracking the temporal dependence of the magnetisation. An exemplary set of measurement results is presented in Fig. 2. We conducted 15 measurements, each lasted longer than 10 hours: 8 measurements on alive cockroaches and 7 on the dead ones, see Supplementary Information (SI) for experimental data. Additionally, more than 10 shorter experiments were conducted (2–5 hours) confirming the trends described below, but are excluded from statistical analysis due to different experimental conditions.

All insects produced measurable magnetic fields. Seven out of eight alive cockroaches gave rise to exponential magnetic field decay with an average decay time of 50 ± 28 minutes. For one alive cockroach we observed a weak stable-in-time signal and this dataset is not included in the average calculation. For another alive cockroach we observed magnetisation revival after the exponential decay, see SI. All measured dead cockroaches gave rise to a stable in time magnetic field similar to data presented in Fig. 2. The decay times have the average of 47.5 ± 28.9 hours.

**Figure 3:**
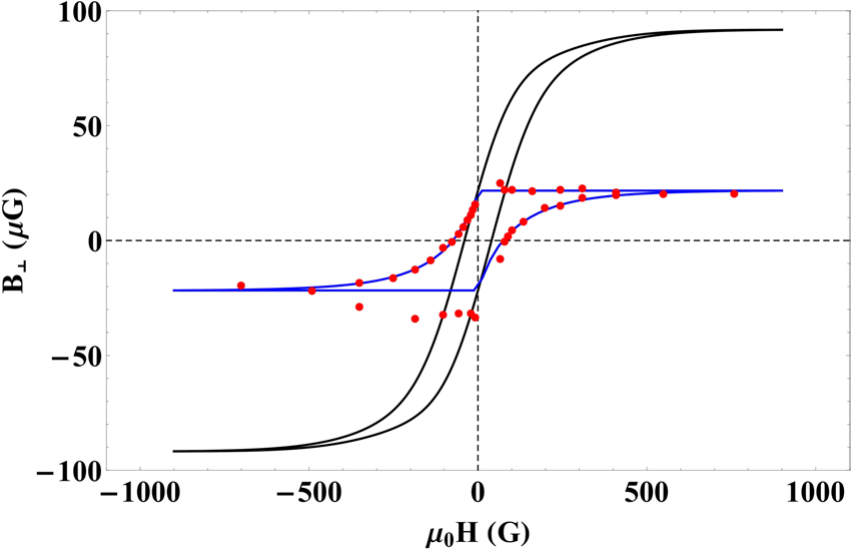
Hysteresis measurements on dead cockroach. The red dots show measured data of remanent magnetic field as a function of applied magnetic field. The blue curve represents a fit to the data obtained with the software package HysterSoft^41^ using the Preisach model. The black curve is an exemplary hysteresis giving rise to our measured remanent hysteresis curve.

In order to gain further insight into the origin of the observed magnetism we conducted a remanent hysteresis measurement the results of which are presented in Fig. 3. Note that this measurement is conducted on a single cockroach only. It motivates our discussion on a possibility of greigite-based magneto-reception in cockroaches.

We model the observed decays with Brownian rotations of magnetic materials. In the Methods section we describe a model that takes into account volume distribution of the magnetic deposits. All its predictions are here explained in simple terms by assuming that observed magnetic relaxation curves match the exponential decay *M*_0_exp(−*t*/*τ*) + offset, where *M*_0_ and *τ* are experimentally determined initial magnetic field and decay time respectively. For Brownian motion of a spherical grain we have:

1 $$\tau =\frac{3V\eta }{{k}_{B}T},$$

where *V* is the effective hydrodynamic volume of a deposit representing the whole sample, *η* viscosity of its environment, *T* denotes the environment’s temperature and *k*_*B*_ Boltzmann constant.

Since no change is expected in the volume of the magnetic materials inside alive and dead insect, Eq. (1) predicts that ratio of the decay times from dead and alive specimen is given by *τ*_*d*_/*τ*_*a*_ = *η*_*d*_/*η*_*a*_, i.e. the ratio of respective viscosities. Hence, the variation in the decay times from alive and dead cockroaches is explained by post-mortem increase in viscosity of the environment of the magnetic particles. From measured data, the viscosity increment is by two orders of magnitude.

The data in Fig. 2 shows different values of the initial magnetic field of alive and dead insect that of course must also be explained by the model. At the end of the magnetisation stage the magnetic field originating in the dead insect can only be smaller than the field generated at this stage by alive insect. There are two contributions diminishing the field: (i) Since the cockroaches were washed in an ultrasonic bath after death, some external magnetic materials could be cleaned; (ii) The increased viscosity may cause partial alignment of internal magnetic deposits with the external field. We first consider the case where the difference in the produced magnetic field is solely from contribution (i). The ratio of the initial magnetic fields, *r* = *M*_0_(dead)/*M*_0_(alive), is now just another variable in the model that characterises the amount of magnetic materials washed out. In this case experimental data does not uniquely specify all the parameters of the model, but only fixes the product *Vη* according to Eq. (1). The second case is more interesting from a theoretical perspective. Assume that there were no external magnetic materials so that contribution (i) can be ignored. In the Methods section we show that partial alignment in highly viscous environment is characterised by the alignment time which only depends on the viscosity, see Eq. (4). In this case experimental data uniquely determines parameters of the model. Their orders of magnitude can be estimated as follows. For the effect of partial alignment, the alignment time *t*_⊗_ in Eq. (4) has to be comparable with 20 minutes as this is how long the cockroaches were magnetised. Assuming they contain magnetic materials similar to magnetite or greigite, with saturation magnetisation 10^5^ A/m, Eq. (4) predicts the viscosity of dead insects  ${\eta }_{d}\sim {10}^{7}$ Pa sec. Accordingly, the viscosity of alive insects is ${\eta }_{a}\sim {10}^{5}$ Pa sec. The hydrodynamic volume then follows from Eq. (1) and the estimated radius of magnetic deposits is 10–100 nm.

 Although magnetic field of dead insects at the end of the magnetisation stage is smaller than the magnetic field of alive insects, the ratio of initial magnetisations r measured in our setup could be higher than 1.This is due to two-minute delay caused by loading the cockroach into magnetometer during which magnetisation of alive insect might already drop below the magnetisation of the dead specimen.According to the model above one finds $r\lesssim 1+2/{\tau }_{a}$, where *τ*_*a*_ is in minutes. Not all of our datasets satisfy this bound as clearly visible in Fig. 2a. This is an indication of underlying multimodal volume distribution. Indeed, the curves in Fig. 2a could be fit with a bimodal distribution with some particles exceeding 100 nm radius, which intuitively can be understood as follows. The small particles decay faster and this combined with the effect of the large viscosity difference leads to the big discrepancy between initial magnetisations of alive and dead insects (taking into account that it takes 2 mins to load the cockroach into the magnetometer). However, this decay is too fast to match the experimental decay times and therefore larger particles have to be introduced.

## Discussion

We showed that partial alignment of magnetic deposits requires a very high viscosity on the order 10^5^ Pa sec in alive cockroaches. Quite high viscosity also follows in the case where some magnetic materials are washed out. Assuming that cockroaches contain single-domain magnetic crystals of radius 50 nm, experimental data shows that they would rotate in an environment of viscosity ~10^2^ Pa sec in alive insect. The obtained high values of viscosity agree with estimations for cytoskeleton inside cells, which was suggested to be a glassy material^28^.

In this context we note that cockroaches may also contain magnetic particles surrounded by a very low viscosity medium. The time resolution of our experiment is set by the time it takes to load a cockroach into the magnetometer, about 2 minutes. Therefore, particles with a relaxation time on the order of tens of seconds are unnoticed. Eq. (1) then shows that in a low viscosity, water-like environment spherical particles up to a micron in radius are not detected.

The increment in viscosity of the environment inside alive and dead cockroaches follows from the expected physiological changes. The dead cells permanently dehydrate causing an increase in volume fraction of cytoskeleton which in turn increases environmental viscosity. The increase in viscosity has already been independently observed inside dying cells^29^.

We note that in the case of partial alignment the estimated size of magnetic materials is in the range of biogenic clusters of magnetic particles that were extracted from two species of termites^25^. This might not be a coincidence as termites are eusocial cockroaches^30^. They belong to the same order Dictyoptera and have closely related symbionts. Nevertheless, we emphasise that our experiments do not prove biogenic magnetism because environmental ferromagnetic contaminants could still be present in the tissues^31^.

The hysteresis measurement (see Fig. 3) reveals a small value of the coercive field, 40 ± 6 G. This is consistent with the measurement on termite *Neocapritermes opacus*^32^. The value is typical for multi-domain materials, ultrafine-grained particles of magnetite^33^ or single-domain greigite^34^, but not single-domain magnetite found in various other animals for which the corresponding coercive field is about 400 G^35^. We pursue further the hypothesis of single-domain greigite. Ref.^36^ identified a black region in the cockroach, *Eublaberus posticus*, hindgut rich in metal sulphides. Unknown organisms producing H_2_S were also found in the flora around the black band. Greigite could form there naturally by reduction of iron in H_2_S rich environment with low oxygen level. Since guts are well-nerved one can speculate that cockroaches could sense magnetic field based on greigite deposits connected to nerves in their hindgut. See refs.^18,20,37–39^ for support of insect magneto-reception through abdomen.

Some evidence already exists for magneto-reception in cockroaches. Cockroach mobility increases when it is placed in an Earth-strength magnetic field (~0.5 G) with periodically varying direction^4^ even after ablation of antennae^5^. This effect is absent under very interesting conditions: (i) in total darkness;^7^ (ii) when a radio-frequency field is added on top of the periodically varying Earth-strength magnetic field;^6^ (iii) in genetically modified cockroaches without Cry2 cryptochromes^7^. Furthermore, the effect depends on light intensity and wavelength^7^ providing support to the hypothesis that cockroaches utilise the so called radical-pair model as basis for their magneto-reception. We now add another piece of evidence supporting this hypothesis. A single-domain greigite particle of radius 50 nm has the magnetic energy in the Earth’s magnetic field almost 100 times above the thermal energy at room temperature and hence it tends to align with the field. However, its alignment time in Earth’s magnetic field would be in the order of hours in a 10^5^ Pa sec viscosity environment (Estimated under the assumption that no external magnetic contaminants are present  on the measured cockroaches). To the contrary, the period of the Earth-strength magnetic field variation in the described behavioural experiments is only 10 minutes.

In conclusion, we realised a non-invasive method of magnetic field measurements applicable to biological systems at physiological temperatures. The method employs an atomic magnetometer with periodically moved sample. Our setup can also be used to infer other physical quantities than magnetic fields as illustrated with the determination of viscosity and volumes using the theory of Brownian motion (magnetorelaxometry). When applied to American cockroaches the method clearly discriminates between dead and alive insects. We propose that its physical origin lies in the post-mortem change of the viscous intracellular environment surrounding magnetic particles. More generally, this technique can be used to test physiological processes or gain insights into the study of stochastic processes driving the motion of biomolecules or magnetic tracer substances inside cells. Independent of any potential magneto-receptive suitability, the characterisation of physical properties of magnetic particles found in biological tissues of various species, even in the human brain^40^, is worth studying. It may help to establish their physiological purpose, which is largely unknown, and to distinguish biogenic precipitates from magnetic contaminants. Our work also highlights the rarely addressed aspect of magnetisation dynamics, which poses limits on the usefulness of biomagnetic ferrites for navigational purposes. Static values of remanence and coercive field strength of magnetic particles are not by themselves sufficient to allow conclusions about magnetosensory suitability, the influence of their surrounding has to be taken into account.

## Methods

### Experimental procedure and data collection

**Figure 4:**
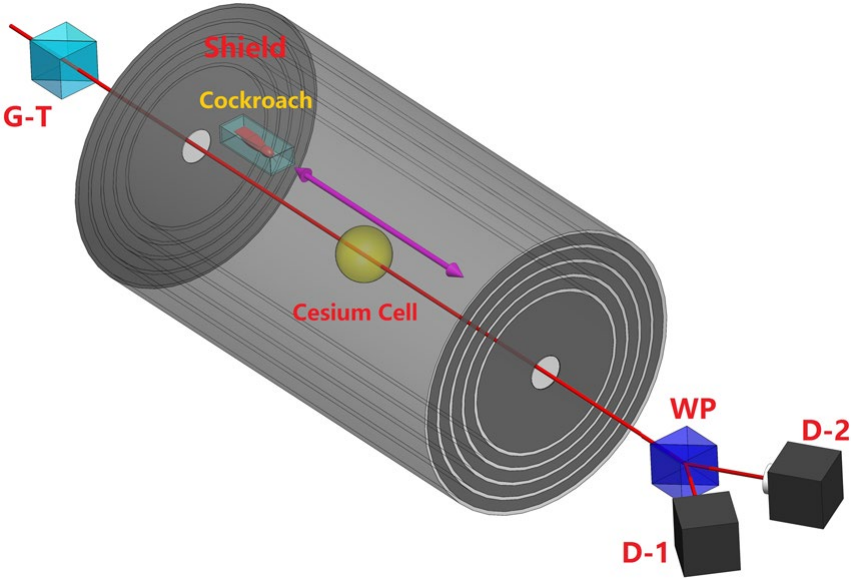
Experimental setup. Only elements essential to the current experiment are shown. See ref.^42^ for the details for a similar setup. Linear polarised light from a Glan-Thomson polariser (G–T) is coupled into a paraffin coated Cs cell. The cell is surrounded by a 5 layer magnetic shield to suppress ambient fields. After the light passes through the cell the induced polarisation rotation is recorded by a Wollaston prism (WP) and balanced detector assembly (D1 and D2). This rotation carries information about the magnetic field at the Cs vapour.

Alive adult female and male cockroaches were kept in transparent insectaria with unlimited water and a diet consisting of cat food pallets and photoperiod of 12 light: 12 dark hours. Before the experiment the insectarium was placed in a 4 °C environment in order to immobilise the insects. The experiments on dead cockroaches took place at least 2 days after death and the dead cockroaches were kept in the 4 °C environment to slow down putrefactive processes. The death was induced by a nitrogen gas atmosphere and thereafter the cockroaches were washed in an ultrasonic bath confirming interior origin of the observed magnetic signal. Every immobilised or dead cockroach was placed in a plastic bag which in turn was put in-between two characterised permanent magnetic plates producing a field of 3 kG at the cockroach location. The direction of induced dipole moment is indicated in Fig. 1. After 20 minutes of magnetisation the sample was mounted on a motorised translation stage inside the optical Caesium atomic magnetometer as shown in Fig. 4. We periodically varied the distance between the sample and the Cs cell for at least 10 hours. One period takes 20 seconds. The recorded data-points are obtained by averaging over 20 periods. Each data-point has a magnetic field uncertainty of 0.08 *μ*G. We verified that the bag alone produces a stable magnetic field over the timespan of our experiment (0.38 ± 0.08 *μ*G).

### Measurement of remanent hysteresis

We placed a dead cockroach in between two N52 grade neodymium permanent magnet plates with the applied magnetic field oriented as in Fig. 1. By changing the separation between the plates the magnitude of the magnetic field at the cockroach position was varied. The magnetic field variation over the cockroach body is at most 15%. The magnetisation time is 10 minutes. We start with an external field equal to 750 G and continue along the hysteresis loop.

### Model

#### Relaxation

Assume that the *i*-th magnetic particle has magnetic dipole moment ${\overrightarrow{\mu }}_{i}$. Initially the cockroaches are magnetised along *z* with macroscopic magnetic moment *M*_*z*_ = *N*〈*μ*〉 + *A*, where *N* is the total number of rotating particles, 〈*μ*〉 is the mean dipole moment and *A* is an offset value due to non-rotating magnetic materials. Diffusion causes rotations of the magnetic deposits, and due to the random character of thermal forces components of the macroscopic moment orthogonal to *z* vanish, 〈*M*_*x*_〉 = 〈*M*_*y*_〉 = 0. The *z* component decays as now the *i*-th rotated microscopic moment contributes with the projection *μ*_*i*_ *cosθ*_*i*_ along the *z* axis. It is convenient to first consider the contribution to the average macroscopic moment from the particles with the same volume. For Brownian rotations the average cosine at time *t* is well known and given by the exponential decay: 〈cos*θ*〉 = exp(−*t*/*τ*), where the decay time, *τ* = 1/2*D*_*r*_, is the inverse of the doubled rotational diffusion coefficient. For simplicity we assume spherically shaped rotating deposits and the decay time reads:

2 $${\tau }_{V}=\frac{3V\eta }{{k}_{B}T},$$

where *V* is the hydrodynamic volume of the deposit, *η* viscosity of its environment, *T* denotes the environment’s temperature and *k*_*B*_ Boltzmann constant. Smaller magnetic particles give rise to a faster decay and they also produce a weaker magnetic field as *μ*_*i*_ = *M*_*s*_*V*_*i*_, where *M*_*s*_ is the saturation magnetisation. The macroscopic moment is obtained by additional averaging over the volumes and reads 〈*M*_*z*_〉 = *NM*_*s*_∫*f*(*V*)*V* exp(−*t*/*τ*_*V*_)d*V* + *A*, where *f*(*V*) is the volume distribution. We take the exponential distribution $f(V)=\mathrm{(1/}\bar{V})\exp (-V/\bar{V})$, with $\bar{V}$ being the average volume, and note that similar results are obtained with a log-normal volume distribution. The average macroscopic moment can now be integrated to the closed form:

3 $$\langle {M}_{z}\rangle =\frac{2N\bar{\mu }t}{{\tau }_{\bar{V}}}{K}_{2}(2\sqrt{t/{\tau }_{\bar{V}}})+A,$$

where $\bar{\mu }={M}_{s}\bar{V}$ and *K*_2_(*x*) denotes the modified Bessel function of the second kind. Fits of this model are presented in Fig. 2.

#### Alignment

For the alignment process we treat particle’s rotation as overdamped harmonic oscillator. This is a suitable approximation in the highly viscous environment. Thermal effects are neglected due to much higher magnetic interaction energy. The angle between magnetic moment and the external field (counted from the field direction) changes as *θ*_*t*_ = *θ*_0_exp(−*t*/*t*_⊗_), where *θ*_0_ is the initial angle and the alignment time *t*_⊗_ reads (see SI):

4 $${t}_{\otimes }\simeq \frac{6\eta }{{M}_{s}B}.$$

Since unmagnetised cockroaches show no magnetic field, we assume a uniform in space distribution of initial magnetic moments. It follows that at time *t* the distribution of angles is still uniform, but in a smaller range *θ*_*t*_ ∈ [0, *θ*_max_], where *θ*_max_ = *π*exp(−*t*/*t*_⊗_). This leads to the *z* component of the macroscopic dipole moment being diminished by a factor of ${cos}^{2}({\theta }_{max}\mathrm{/2)}$ as compared to the value when all magnetic moments are aligned. In our experiments, the cockroaches were magnetised for 20 minutes and hence we use this number for *t* in *θ*_max_. The saturation magnetisation is chosen as *M*_*s*_ = 3 × 10^5^ A/m, with the order of magnitude matching magnetite and greigite. After magnetisation it then takes 2 minutes to mount the cockroach container in the magnetometer during which Brownian rotations cause partial demagnetisation according to Eq. (3). At this stage we fit the model to the observed data. We first fit measurement results from alive insect and then vary only viscosity to fit the data from dead cockroach.

## Electronic supplementary material

Supplementary information

## References

[1] Cronin TW (2003). Polarization vision and its role in biological signaling. Integr. Comp. Biol..

[2] Wiltschko W, Wiltschko R (2005). Magnetic orientation and magnetoreception in birds and other animals. J. Comp. Physiol. A.

[3] Lambert N (2012). Quantum biology. Nat. Phys..

[4] Vacha M (2006). Laboratory behavioural assay of insect magnetoreception: magnetosensitivity of periplaneta americana. J. Exp. Biol..

[5] Vacha M, Puzova T, Drstkova D (2008). Ablation of antennae does not disrupt magnetoreceptive behavioural reaction of the american cockroach to periodically rotated geomagnetic field. Neurosci. Lett..

[6] Vacha M, Puzova T, Kvicalova M (2009). Radio frequency magnetic fields disrupt magnetoreception in american cockroach. J. Exp. Biol..

[7] Bazalova O (2016). Cryptochrome 2 mediates directional magnetoreception in cockroaches. Proc. Nat. Acad. Sci..

[8] Vacha M, Kvicalova M, Puzova T (2010). American cockroaches prefer four cardinal geomagnetic positions at rest. Behaviour.

[9] Camplitepe Y, Aksoy V, Neslihan U, Ayse Y, Becenen I (2005). An experimental analysis on the magnetic field sensitivity of the black-meadow ant formica pratensis retzius (hymenoptera: Formicidae). Acta Biol. Hung..

[10] Wajnberg E (2010). Magnetoreception in eusocial insects: an update. J. Roy. Soc. Inter..

[11] Lindauer M, Martin H (1968). Die Schwereorientierung der Bienen unter dem Einfluss des Erdmagnetfeldes. Z. Vgl. Physiol..

[12] Gould JL, Kirshvink JL, Deffeyes KS, Brines ML (1980). Orientation of demagnetised bees. J. Exp. Biol..

[13] Walker MM, Bitterman ME (1985). Conditioned responding to magnetic fields by honeybees. J. Comp. Physiol. A.

[14] Kirschvink JL, Kobayashi A (1991). Is geomagnetic sensitivity real? Replication of the Walker-Bitterman magnetic conditioning experiment in honey bees. Amer. Zool..

[15] Walker MM, Bitterman ME (1989). Honeybees can be trained to respond to very small changes in geomagnetic field intensity. J. Exp. Biol..

[16] Kirschvink JL, Padmanabha S, Boyce CK, Oglesby J (1989). Measurement of the threshold sensitivity of honeybees to weak, extremely low-frequency magnetic fields. J. Exp. Biol..

[17] Frier HJ, Edwards E, Smith C, Neale S, Collett TS (1996). Magnetic compass cues and visual pattern learning in honeybees. J. Exp. Biol.

[18] Gould JL, Kirshvink JL, Deffeyes KS (1978). Bees have magnetic remanence. Science.

[19] Kuterbach DA, Walcott B, Reeder RJ, Frankel RB (1986). Iron-containing cells in the honey bee (apis mellifera). Science.

[20] Pan W (2016). Evidence for the presence of biogenic magnetic particles in the nocturnal migratory brown planthopper, nilaparvata lugens. Sci. Rep..

[21] Jones DS, MacFadden BJ (1982). Induced magnetisation in the monarch butterfly, danaus plexippus. J. Exp. Biol..

[22] Acosta-Avalos D (1999). Isolation of magnetic nanoparticles from pachycondyla marginata ants. J. Exp. Biol..

[23] Alves OC, Wajnberg E, Oliveira JFd, Esquivel DMS (2004). Magnetic material arrangement in oriented termites: a magnetic resonance study. J. Magn. Res..

[24] Wajnberg E, Cernicchiaro G, Esquivel DMS (2004). Antennae: the strongest magnetic part of the migratory ant. BioMetals.

[25] Maher BA (1998). Magnetite biomineralization in termites. Proc. R. Soc. Lond. B..

[26] Budker, D. & Jackson Kimball, D. F. *Optical Magnetometry* (Cambridge University Press 2013).

[27] Leliaert J (2017). The complementarity and similarity of magnetorelaxometry and thermal magnetic noise spectroscopy for magnetic nanoparticle characterization. J. Phys. D.

[28] Fabry B (2001). Scaling the microrheology of living cells. Phys. Rev. Lett..

[29] Kuimova MK (2009). Imaging intracellular viscosity of a single cell during photoinduced cell death. Nat. Chem..

[30] Inward D, Beccaloni G, Eggleton P (2007). Death of an order: a comprehensive molecular phylogenetic study confirms that termites are eusocial cockroaches. Bio. Lett..

[31] Kobayashi AK, Kirschvink JL, Nesson MH (1995). Ferromagnetism and emfs. Nature.

[32] Ferreira J (2005). Comparative magnetic measurements on social insects. J. Magn. Magn. Mater..

[33] Geiss CE, Egli R, Zanner CW (2008). Direct estimates of pedogenic magnetite as a tool to reconstruct past climates from buried soils. J. Geophys. Res..

[34] Diaz-Ricci JC, Kirschvink JL (1992). Magnetic domain state and coercivity predictions for biogenic greigite (fe_3_ s_4_): A comparison of theory with magnetosome observations. J. Geophys. Res..

[35] Diebel CE, Proksch R, Green CR, Neilson P, Walker MM (2000). Magnetite defines a vertebrate magnetoreceptor. Nature.

[36] Cruden DL, Gorrell TE, Markovetz AJ (1979). Novel microbial and chemical components of a specific black-band region in the cockroach hindgut. J. Bacteriol..

[37] Walker MM, Bitterman ME (1989). Attached magnets impair magnetic field discrimination by honeybees. J. Exp. Biol..

[38] Liang C-H, Chuang C-L, Jiang J-A, Yang E-C (2016). Magnetic sensing through the abdomen of the honey bee. Sci. Rep..

[39] Lambinet V (2017). Linking magnetite in the abdomen of honey bees to a magnetoreceptive function. Proc. R. Soc. B.

[40] Kirschvink JL, Kobayashi-Kirschvink A, Woodford BJ (1992). Magnetite biomineralization in the human brain. Proc. Nat. Acad. Sci..

[41] Dimian, M. & Andrei, P. *Noise-Driven Phenomena in Hysteretic Systems*, vol. 218 of *Signals and Communication Technology* (Springer, New York 2014).

[42] Crepaz H, Ley LY, Dumke R (2015). Cavity enhanced atomic magnetometry. Sci. Rep..

