# Supplementary material for: In-vivo biomagnetic characterisation of the American cockroach
Source: Sci Rep. 2018 Mar 23;8:5140. doi: 10.1038/s41598-018-23005-1 (PMC5865160; doi:10.1038/s41598-018-23005-1)
Supplement: Supplementary file 1 — Supplementary information [file 41598_2018_23005_MOESM1_ESM.pdf]

In-vivo biomagnetic characterisation of the American cockroach
Supplementary Information

Ling-Jun Kong,[1,2] Herbert Crepaz,[1,3] Agnieszka Górecka,[1,4]
Aleksandra Urbanek,[5] Rainer Dumke,[1,3] and Tomasz Paterek[1,3]

[1] *School of Physical and Mathematical Sciences, Nanyang Technological University, 637371 Singapore*
[2] *MOE Key Laboratory of Weak Light Nonlinear Photonics and School of Physics, Nankai University, Tianjin 300071, China*
[3] *Centre for Quantum Technologies, National University of Singapore, 117543 Singapore*
[4] *School of Physics and Astronomy, Monash University, Victoria 3800, Australia*
[5] *Department of Invertebrate Zoology and Parasitology, University of Gdańsk, 80-308 Poland*

I. MEASURED DATA

Tables below list experimental data used in statistical analysis of the main text.

Alive

Dataset No	Cockroach No	Decay Time [min]	Initial Magnetic Field [Gauss]
1	1	25	7
2	2	22	1
3	3	89	0.54
4	4	71	1.8
5	4	77	1.1
6	5	30	0.71
7	6	37	0.71

Dead

Dataset No	Cockroach No	Decay Time [min]	Initial Magnetic Field [Gauss]
8	1	4956	17
9	2	1374	0.77
10	4	2178	1.2
11	4	2178	1.05
12	5	2136	0.5
13	5	5676	0.8
14	5	1440	0.61

II. FASTED COCKROACH

We verified that the food pallets given to cockroaches can be magnetised and therefore conducted the experiment with fasted cockroach in order to exclude the hypothesis that the observed decay is due to ingested magnetism of the food. The mean food transit time was determined to be 20.6 hours, with a part of each meal retained in the crop for up to 4 days [1]. We therefore fasted the cockroach for 7 days, giving it only water, and found that this had no effect on the decaying magnetic field. This is not a conclusive proof of biogenic magnetism because environmental ferromagnetic contaminants could still be present in the tissues [2].

III. MAGNETIC FIELD REVIVAL

The theory outlined in the main text explains exponential magnetic field decay which was observed for most cockroaches. For this one insect, however, we have seen a revival of the magnetic field as shown in the figure.

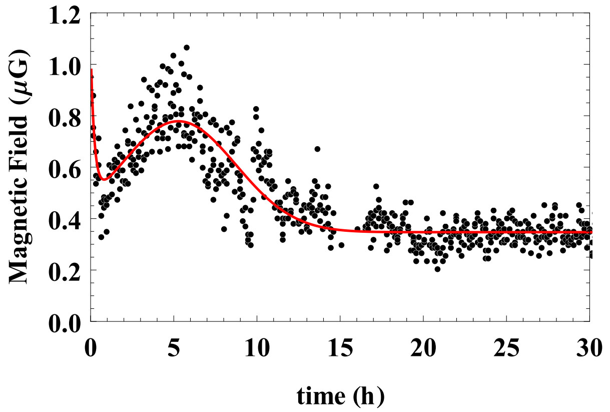

IV. ALIGNMENT TIME

Consider a spherical particle endowed with magnetic moment $\vec{\mu}$ and surrounded by an environment with viscosity η at room temperature T. If the particle is subject to an external magnetic field $\vec{B}$, its rotational motion is described by the Newton law:

$$I\ddot{\theta} = -f\dot{\theta} - \mu B \sin\theta + \mathcal{T}. \tag{1}$$

Here θ denotes the angle between $\vec{B}$ and $\vec{\mu}$ (counted from $\vec{B}$), I stands for the moment of inertia of the sphere, $I = \frac{2}{5}\rho V R^2$, and f is the rotational friction coefficient, $f = 8\pi\eta R^3$. The next term gives magnetic torque and the last term is the thermal torque whose influence we will ignore here because the strong aligning field gives rise to $\mu B \gg kT$ even for very small magnetic moments. Despite its simplicity, for particles embedded in a highly viscous environment subjected to a constant field, similar models have successfully explained experimental results [3].

Our aim is to calculate the time it takes the particle to align with the field, $t_\otimes$. Note that $t_\otimes$ is longer than the alignment time $t_\downarrow$ obtained when the magnetic torque is replaced by a stronger torque. Similarly, $t_\otimes$ is shorter than the alignment time $t_\uparrow$ obtained if the magnetic torque is replaced by a weaker torque. We show a simple upper and lower bound on the strength of the magnetic torque leading to alignment times that differ only by a constant factor of order one. Hence the obtained formula also holds for $t_\otimes$.

Consider first the stronger torque $\mu B \sin\theta \le \mu B\theta$. The original nonlinear problem now reduces to the damped harmonic oscillator. Due to the assumed high viscosity $f^2 - 4I\mu B \gg 0$, the oscillation is overdamped with the particular solution

$$\theta_t = \left(\theta_0 - \frac{r_-\theta_0}{r_- - r_+}\right)\exp(r_- t) + \frac{r_-\theta_0}{r_- - r_+}\exp(r_+ t), \tag{2}$$

where

$$r_\pm = \frac{1}{2}\left(-\frac{f}{I} \pm \sqrt{\frac{f^2}{I^2} - 4\frac{\mu B}{I}}\right). \tag{3}$$

The initial conditions are: $\theta(0) = \theta_0$ for the angle and $\dot{\theta}(0) = 0$ for the angular velocity. Since $(f/I)^2 \gg 4\mu B/I$ we simplify:

$$r_+ = -\frac{\mu B}{f}, \tag{4}$$

$$r_- = -\frac{f}{I} + \frac{\mu B}{f}. \tag{5}$$

Furthermore, both $r_\pm$ are negative with $r_- \ll r_+$ and therefore $\exp(r_- t)$ quickly decays to zero. The long time dynamics is governed by the decay $\exp(r_+ t)$, which admits alignment time:

$$t_\downarrow = \frac{f}{\mu B} = \frac{6\eta}{M_s B}. \tag{6}$$

Note that this time is independent of the volume of the particle as well as its initial angle.

For the estimation of $t_\uparrow$ consider the magnetic moment at the initial angle θ_0. Since the torque tends to align it with the field, the accessible angles are from θ_0 to 0. This gives rise to the following lower bound: $\mu B \frac{\sin\theta_0}{\theta_0}\theta \leq \mu B \sin\theta$. The problem reduces to the damped harmonic oscillator as above with the replacement $\mu B \to \mu B \frac{\sin\theta_0}{\theta_0}$. Assuming uniform initial distribution of magnetic moments the average alignment time is:

$$t_\uparrow = \frac{1}{4\pi}\int_0^\pi \int_0^{2\pi} d\varphi d\theta_0 \sin\theta_0 \frac{6\eta\theta_0}{M_s B \sin\theta_0} = \frac{\pi^2}{4}\frac{6\eta}{M_s B}.$$

Hence both upper and lower bounds on the average alignment time are size independent and of the same order of magnitude as $\frac{\pi^2}{4} \approx 2.5$.

[1] B. T. Snipes and O. E. Tauber, Ann. Ent. Soc. Am. **30**. 277 (1937).
[2] A. K. Kobayashi, J. L. Kirschvink, and M. H. Nesson, Nature **374**, 123 (1995).
[3] J.-C. Eloi, M. Okuda, S. E. Ward Jones, and W. Schwarzacher, Biophys. J. **104**, 2681 (2013).
